# Benchmarking Nutraceutical Soybean Composition Relative to Protein and Oil

**DOI:** 10.3389/fnut.2021.663434

**Published:** 2021-08-11

**Authors:** Constanza S. Carrera, Fernando Salvagiotti, Ignacio A. Ciampitti

**Affiliations:** ^1^Unidad de Estudios Agropecuarios, Consejo Nacional de Investigaciones Científicas y Técnicas (CONICET), Instituto Nacional de Tecnología Agropecuaria (INTA), Córdoba, Argentina; ^2^Department of Agronomy, 2004 Throckmorton Plant Science Center, Kansas State University, Manhattan, KS, United States; ^3^Consejo Nacional de Investigaciones Científicas y Técnicas (CONICET), Buenos Aires, Argentina; ^4^Crops, Soil and Water Management Group, Estación Experimental Agropecuaria Instituto Nacional de Tecnología Agropecuaria (INTA) and Consejo Nacional de Investigaciones Científicas y Técnicas (CONICET), Oliveros, Argentina

**Keywords:** total isoflavone, total tocopherol, soybean seed composition, seed weight, nutraceutical compounds

## Abstract

The aim of this study was to explore relationships between protein, oil, and seed weight with seed nutraceutical composition, focused on total isoflavone (TI) and total tocopherol (TT) contents across genotypic and environmental combinations in soybean. We conducted a synthesis-analysis of peer-reviewed published field studies reporting TI, TT, protein, oil, and seed weight (*n* = 1,908). The main outcomes from this synthesis-analysis were: (i) relationship of TI-to-protein concentration was positive, though for the upper boundary, TI decreases with increases in protein; (ii) relationship of TT-to-oil concentration was positive, but inconsistent when oil was expressed in mg per seed; and (iii) as seed weight increased, TI accumulation was less than proportional relative to protein concentration and TT decreased more proportional relative to oil concentration. Association between nutraceuticals and protein, oil, and seed weight for soybean reported in the present study can be used as a foundational knowledge for soybean breeding programs interested on predicting and selecting enhanced meal isoflavone and/or oil tocopherol contents.

## Introduction

Seed protein and oil in soybeans [*Glycine max* (L.) Merrill] define the overall quality value for the international trade markets (1). Additionally, more recently, soybean has ranked as one of the top sources of highly valuable nutraceutical compounds with health-enhancing properties (2). These active substances extracted from plants origin (phytocomplexes), such as soybean seeds, are important given their proven efficacy and benefits on human health (for prevention or support treatment of some pathologic scenarios) in addition to their nutritional content (3–5). For such relevant properties, these compounds are usually referred to as nutraceuticals (6). From the nutraceutical seed components, isoflavones (minor components of meal) plays a key role in the prevention and treatment of chronic diseases (e.g., cancer, heart disease, osteoporosis) (7) due to its anti-estrogenic and antioxidant activities (8). Several health benefits have been associated with tocopherols, minor components of oil with lipophilic antioxidant properties, playing a critical role in delaying the pathogenesis of cardiovascular and neurodegenerative diseases (e.g., Alzheimer's and Parkinson's) (9).

In the past two decades, several studies have been conducted to quantify the variation in soybean seed composition relative to the interaction of genotype by environment (G × E) (10–20). Breeding programs aimed at improving soybean compositional quality, i.e., increasing the content of key nutrients such as isoflavones (21), have invested considerable efforts in estimating correlations between seed components for different G × E (22–24). Past research studies have already reported trade-offs for protein and oil (25–27), isoflavone and protein (23, 28, 29), and tocopherol and oil weight per grain (30), but with the latter association receiving considerably less attention in the scientific literature.

Seed size defined by weight of seeds, is an important character for different type of soybean foods. Protein, oil, isoflavones, and tocopherols deposition patterns mimic seed dry mass accumulation during seed filling period in soybean (1). Thus, final seed weight may act as an integrative indicator of ecophysiological processes occurring during this period when these seed components accumulate. However, association of nutraceuticals with seed weight has not been comprehensively analyzed. Those relationships may be tightly linked to the particular genotypic and environmental combinations explored in each study, making difficult to assess the biological limits of nutraceuticals composition relative to protein and oil. Thus, a synthesis-analysis, by quantitatively combining results from different studies, may overcome those limitations, expanding the level of inference as well as increasing the validity of conclusions (31, 32). A similar approach has been previously explored for analyzing relationships between grain yield and a given resource such as water availability (33) or nutrient (N, P, and K) (34) in wheat or in soybean (35–37).

Several review studies have been published to synthesize knowledge on soybean seed composition (6, 38–41). However, until the present a more comprehensive characterization of the variation for seed nutraceuticals with relevant agronomic traits under diverse genotypic and environmental combinations is still lacking. Therefore, the aim of this study was to explore relationships between protein, oil, and seed weight with seed nutraceutical composition, focused on total isoflavone (TI) and total tocopherol (TT) contents across different genotypic and environmental combinations in soybean.

## Materials and Methods

### Database Compilation and Variables Evaluated

The literature search for the database compilation was conducted by surveying peer-reviewed journal articles published over the past decades following the procedures described in previous review papers (35, 42). Briefly, collected studies were evaluated under field conditions reporting total isoflavone (TI) and total tocopherol (TT) contents (expressed as mg and μg component per seed in dry basis, respectively), protein and oil concentrations (expressed as percentage of dry weight) and/or contents (mg component per seed in dry basis) as well. Seed protein and oil concentrations from all studies were adjusted to a standard moisture basis of 130 g kg^−1^, because wet basis moisture content is generally used for industrial and commercial purposes (43). Only two studies were meeting the criteria for being included in this review i.e., that evaluated the four above-mentioned seed chemical traits (20, 22), yielding insufficient data to address the objectives of our work. Thus, two databases were generated, one comprising protein and TI, involving 12 studies (Table 1) from Argentina (3), Brazil (2), Canada (4), Korea (1), and the United States (US) (2) and the second database comprising oil and TT, and involving 5 studies (Table 2) from Argentina (1), China (1), and US (3). Since not all studies reported simultaneously the seed weight, the number of cases for protein and oil concentrations and contents in each database were different. For instance, database 1 includes 1,624 data points for protein concentration and total isoflavone, and 1,600 data points for protein content. Database 2 includes 284 data points for oil concentration and total tocopherol, and 255 data points for oil content. In all cases, the databases included studies primarily focused on quantifying soybean seed composition as affected by G × E combinations. Data were retrieved directly from tables or digitalized from figures.

**Table 1:** Number of study, country, latitude, number of involved environments defined as crop year, location, and sowing date combinations, author and year of publication, experimental conditions and design, cropping season, treatments evaluated, and genotype type for each study included in the soybean seed protein and isoflavone meta-data base.

**N°**	**Country**	**Latitude**	**Environments**	**Author, year**	**Experimental design**	**Cropping season**	**Treatments**	**Genotype**
1	Argentina	31° 49′S, 63° 46′W	16	Carrera et al. (44)	Randomized complete-block design with three replications	2016–2017 2017–2018	Level and timing of light interception reduction	Transgenic GM IV
2	Argentina	31° 10′S, 61° 28′W to 38° 19′S, 60° 14′W	37	Carrera et al. (15), Carrera and Dardanelli (45)	Randomized complete-block design with three replications	2001–2002 2003–2004	Temperature × water deficit during seed filling	Transgenic GM III, IV, V, VI, VII, VIII
3	Argentina	27° 04′S, 65° 25′W to 38° 19′S, 60° 14′W	15	Carrera et al. (11)	Randomized complete-block design with three replications	2006–2007 2007–2008	Genotype and environment, and their combination	Non-transgenic GM IV, IV, V high protein types high oil types
4	Brazil	23° 25′S, 51° 56′W and 23°45′S, 53°19′W	2	Rizzatti Ávila et al. (46)	Randomized complete-block design with four replications	2004–2005	Sowing dates × locations	Non-transgenic GM VI and VII
5	Brazil	17° 13′S, 46° 52′W and 19° 18′S, 46° 02′W	2	Oliveira et al. (47)	Randomized complete-block design with three replications	2001–2002	Sowing dates × locations	Non-transgenic GM VII-VIII high protein types high oil types
6	Canada	45° 25′N, 73° 56′W	6	Al-Tawaha et al. (48)	Randomized complete-block design with split-plot restriction and three replications	2003 and 2004	Genotype and irrigation	Non-transgenic MG 0–000 Food grade
7	Canada	45° 23′N, 75° 43′W	12	Morrison et al. (23)	Randomized complete-block design with four replications	1993–2004	Genotype and environment	Non-transgenic MG 0–00 Food grade
8	Canada	56° 07′N, 106° 20′W	Not reported	OSCC (49). Ontario Soybean And Canola Committee, 2005–2018	Not reported	2005–2018	Genotype and environment	Non-transgenic MG 00–II Food grade
9	Canada	51° 15′N, 85°19′W	12	Yin and Vyn (50)	Randomized complete-block design with four replications	1998–2000	Nutrient managment × yield level	Non-transgenic and transgenic MG 0 and I
10	Korea	35° 30′N, 128° 44′E	1	Lee et al. (51)	Not reported	2013	Biochemical characterization (192 Korean germplasm accessions)	Not reported
11	USA	40° 37′N, 89° 23′W	1	Harrigan et al. (20)	Randomized complete-block design with six replications	2011	Genotype and environment	Non-transgenic and transgenic GM not reported
12	USA	33°26′N, 90°55′W	8	Bennett et al. (52)	Randomized complete-block design with four replications	2002–2003	Management practices (planting date and irrigation)	Transgenic MG IV and V

**Table 2:** Number of study, country, latitude, number of involved environments defined as crop year, location, and sowing date combinations, author and year of publication, experimental conditions and design, cropping season, treatments evaluated, and genotype type for each study included in the soybean seed oil and tocopherol meta-data base.

**N°**	**Country**	**Latitude**	**Environments**	**Author, year**	**Experimental design**	**Cropping season**	**Treatments**	**Genotype**
1	Argentina	27° 04′S, 65° 25′W to 38° 19′S, 60° 14′W	15	Carrera et al. (15)	Randomized complete-block design with three replications	2006–2007 to 2007–2008	Environmental correlation between seed chemical compounds	Non-transgenic GM: IV, IV, V high protein types high oil types
2	China	26° 14′N, 119° 36′E to 47° 07′N, 128° 44′E	8	Yu et al. (53)	Not reported	Not reported	Genotype	Non-transgenic and transgenic GM not reported
3	USA	40° 37′N, 89° 23′W	1	Harrigan et al. (20)	Randomized complete-block design with six replications	2011	Genotype and environment	Non-transgenic and transgenic GM not reported
4	USA	41° 29′N, 93° 29′W to 42° 01′N, 93° 37′W	3	Scherder et al. (54)	Randomized complete-block design with two replications	2004	Genotype and environment	Reduced and normal palmitate lines GM not reported
5	USA	39° 02′N, 76° 38′W	3	Whent et al. (19)	Not reported	2007	Genotype, environment, and their interaction	Reduced and normal linolenic genotypes GM not reported

### Database Analysis

Total isoflavone and total tocopherol contents, as well as protein and oil concentrations and contents from both databases were analyzed using descriptive statistics: number of observations (*n*), mean, standard deviation (SD), median, range of variation (minimum and maximum), quartile 25% as well as 75%, i.e., interquartile range (IQR), with 50% of all observations centered around the median. Analyses were complemented with histograms to visualize the distributions of seed components (GraphPad Prism version 7.0). Seed total isoflavone-to-protein and seed total tocopherol-to-oil relationships were modeled using quantile regression techniques (55, 56). Envelopes portraying maximum (0.99 quantile) and minimum (0.01 quantile) boundaries, and the envelopes enclosing 50% of all the observed data, namely the IQR as described in Salvagiotti et al. (57) were obtained using Blossom software (58). In addition, total isoflavone-to-protein and total tocopherol-to-oil ratios for the boundary functions were further studied via linear and quadratic regressions testing seed weight (mg per seed) as an independent variable for explaining changes in soybean seed composition variation. Model selection was based on the residue analysis and the coefficient of determination (*R*^2^) (59). In case of significant quadratic regressions, the first derivative of the functions that indicates the seed weight at which a trait accumulation rates is zero was analyzed.

## Results

### Variation of Soybean Seed Protein, Total Isoflavone, Oil, and Total Tocopherol

**Figure 1:**
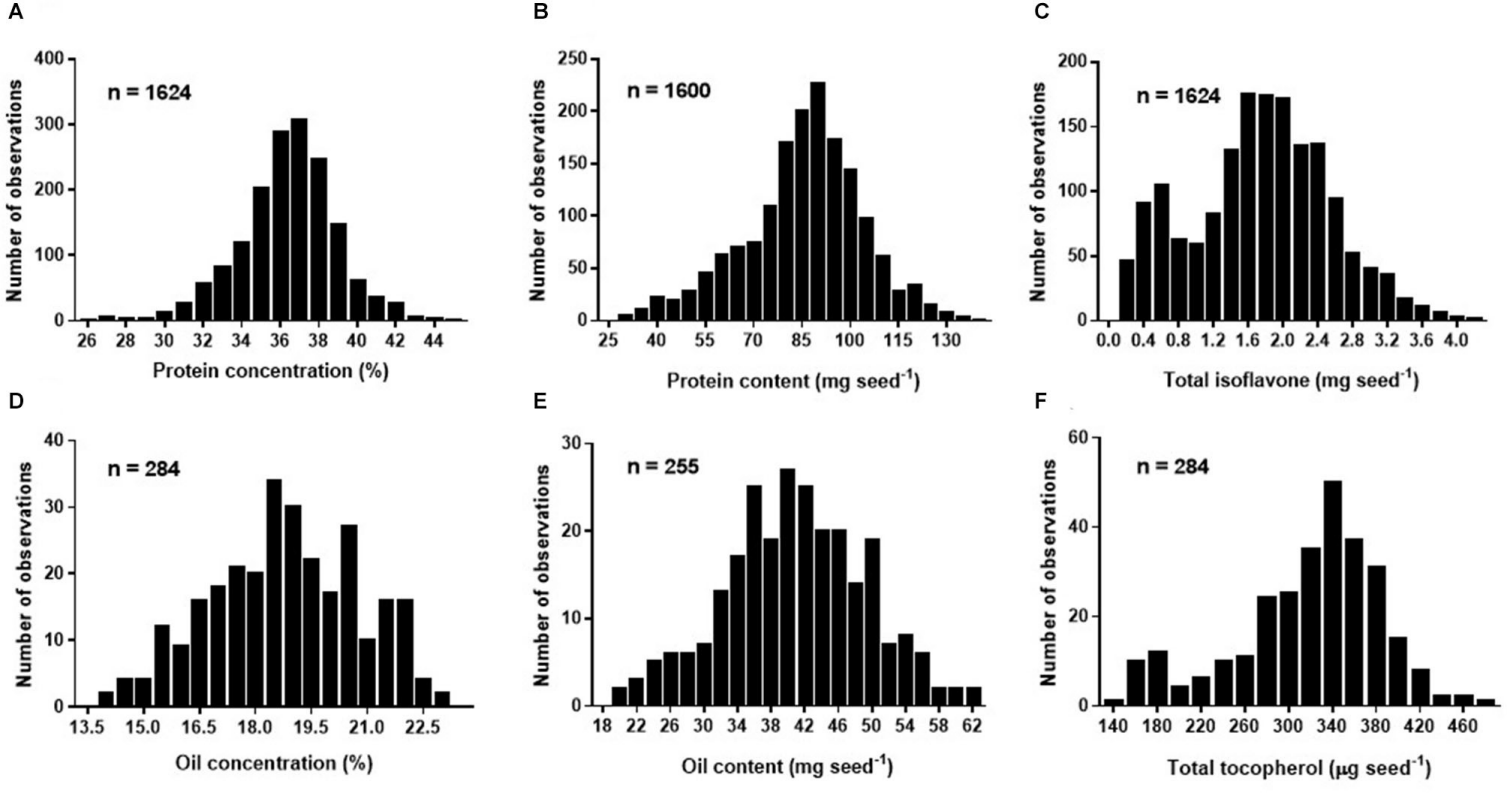
Frequency histogram for soybean seed protein concentration (%) adjusted to 13% moisture **(A)**, protein content expressed in mg seed^−1^ **(B)**, total isoflavone content expressed in mg seed^−1^ **(C)** of Database 1, and oil concentration (%) adjusted to 13% moisture **(D)**, oil content expressed in mg seed^−1^ **(E)**, and total tocopherol content expressed in μg seed^−1^ **(F)** of Database 2.

Overall mean seed protein concentration, content and total isoflavone were 36%, 87 mg seed^−1^, and 1.76 mg seed^−1^, respectively (Table 3). Differences between maximum and minimum values were 72 and 366% for protein concentration (IQR of 35–38%) and content (IQR of 76–97 mg seed^−1^), respectively, and 3,358% for total isoflavone content (IQR of 1.22–2.26 mg seed^−1^). On the other hand, mean seed oil concentration, content and total tocopherol were 19%, 40 mg seed^−1^, and 331 μg seed^−1^, respectively (Table 3). For oil concentration (IQR of 17–20%) and content (IQR of 35–47 mg seed^−1^) differences between maximum and minimum values were 65 and 214%, respectively, and 228% for total tocopherol content (IQR of 283–360 μg seed^−1^). The great variability observed for all the traits can be attributed to the contrasting environments (climate, soil types) and cropping management (e.g., sowing date, plant density, genotypes, water regimes, and tillage) interactions during the seed filling period across the regions. Frequency distributions for protein concentration and content (i.e., database 1) were slightly skewed (−0.33 and −0.37, respectively), with a leptokurtic (kurtosis = 1) and close to a mesokurtic distribution (kurtosis = 0.27), respectively (Figures 1A,B). Total isoflavone was more normally distributed close to a mesokurtic distribution (kurtosis = −0.4) (Figure 1C). Distribution for oil and total tocopherol (i.e. database 2) concentration were slightly skewed (skew = −0.11, −0.73, respectively), while oil content was normally distributed but all three variables exhibiting a slightly flat distribution (kurtosis = −0.58, −0.23, 0.18, for oil concentration, content and total tocopherol, respectively Figures 1D–F).

**Table 3:** Descriptive statistics of the two meta-databases, one relative to soybean seed protein concentration [expressed as percentage of dry weight (DW) and adjusted to 13% moisture] and content (mg seed^−1^), and total isoflavone (TI) content (mg seed^−1^); the other relative to seed oil concentration (expressed as percentage of DW and adjusted to 13% moisture) and content (mg seed^−1^), and total tocopherol (TT) content (μg seed^−1^).

**Data base**	**Variable**	**Unit**	***n***	**Mean**	**SD**	**Minimum**	**25% Q**	**Median**	**75% Q**	**Maximum**
Protein-TI	Protein (DW)	%	1,624	41.8	2.81	30.0	40.2	41.9	43.5	51.6
	Protein (13% moisture)	%	1,624	36.4	2.45	26.1	35.0	36.5	37.8	44.9
	Pr	mg seed^−1^	1,600	85.5	18.2	29.6	76.0	87.2	97.0	138.1
	TI	mg seed^−1^	1,624	1.73	0.79	0.12	1.22	1.76	2.26	4.15
Oil-TT	Oil (DW)	%	284	21.6	2.3	16.1	20.0	21.6	23.3	26.5
	Oil (13% moisture)	%	284	18.8	2.0	14.0	17.4	18.8	20.3	23.1
	Oil	mg seed^−1^	255	40.8	8.4	19.7	35.3	40.4	46.8	61.6
	TT	μg seed^−1^	284	318.2	66.0	143.5	282.8	331.4	360.4	471.0

### Nutraceutical Soybean Composition Relative to Protein and Oil

**Figure 2:**
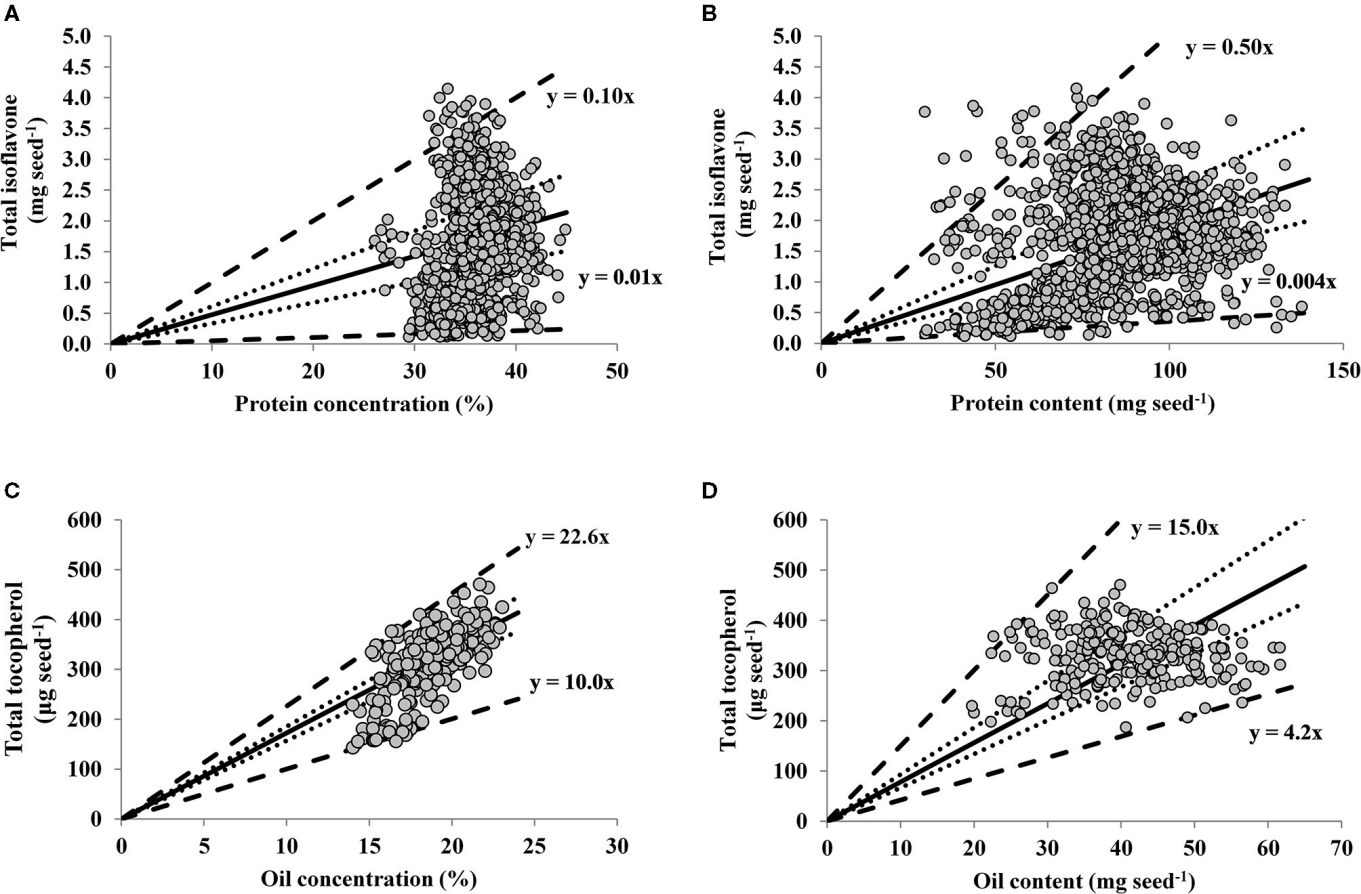
Relationships between seed total isoflavone (mg seed^−1^) vs. protein concentration (%, adjusted to 13% moisture content) **(A)** and vs. protein content (mg seed^−1^) **(B)**; and between soybean seed total tocopherol (μg seed^−1^) vs. oil concentration (%, adjusted to 13% moisture content) **(C)** and vs. oil content (mg seed^−1^) **(D)** across a range of soybean genotypes and environments. In all panels lines represent quantile regression for percentiles 1 and 99 (thick dotted lines) percentiles 25 and 75 (dotted lines). The solid line is the average fit of the data, for which the following models were computed: **(A)** *Y* = −0.37 + 0.06*X*, adjusted *R*^2^ = 0.03, *P* < 0.0001; **(B)** *Y* = 0.81 + 0.01*X*, adjusted *R*^2^ = 0.06, *P* < 0.0001; **(C)** *Y* = −118.93 + 23.27*X*, adjusted *R*^2^ = 0.48, *P* < 0.0001; **(D)** *Y* = 326.0 + 0.15*X*, adjusted *R*^2^ = 0.00, *P* = 0.70.

For the relationships between seed nutraceutical with protein and oil, the slopes of the linear regressions for percentiles 1 and 99 were 0.005 and 0.10 mg TI seed^−1^ % protein^−1^ (Figure 2A), 0.004 and 0.05 mg TI seed^−1^ mg protein seed^−1^ (Figure 2B), and 10.0 and 22.6 μg TT seed^−1^ % oil^−1^ (Figure 2C), 4.2 and 15.0 μg TT seed^−1^ mg oil seed^−1^ (Figure 2D), respectively. Thus, the upper boundary lines represent maximum seed protein (Figures 2A,B) or oil (Figures 2C,D), implying that TI or TT are limited by factors other than protein or oil, respectively. In contrast, the lower boundary lines indicate maximum TI or TT dilution, with protein (Figures 2A,B) or oil (Figures 2C,D) as the main limiting factors for TI or TT, respectively. The distribution of data points in the TI-to-protein concentration relationship (Figure 2A) depicts a positive association up to 35% of protein (13% moisture basis), above which increases in protein concentration seem to be not accompanied by increases in seed TI, plausible portraying the trade-off generally reported between these two traits. Respect to the TT-to-oil concentration relationship, there was not a clear plateauing in seed TT content at high levels of oil concentration (Figure 2C), suggesting that soybean TT accumulation rate per unit of oil remains constant even at high levels of oil concentration (above 20% expressed in 13% moisture basis).

However, when the relationships of both protein and oil are expressed in terms of their contents (i.e., in mg of protein or oil per seed), the TI-to-protein relationship presented a larger variation relative to TT-to-oil content. Indeed, in the range of oil content variation (20–61 mg seed^−1^), TT varied 151%. For the range of protein variation (30–138 mg seed^−1^), TI varied 3,358%, with 22% more variability than TT. This greater variability may explain the significant and positive association for TI and protein content (*p* < 0.0001, Figure 2B), with a non-significant for TT and oil content (*p* = 0.70, Figure 2D).

### Relations of Nutraceuticals, Protein and Oil With Individual Seed Weight

**Figure 3:**
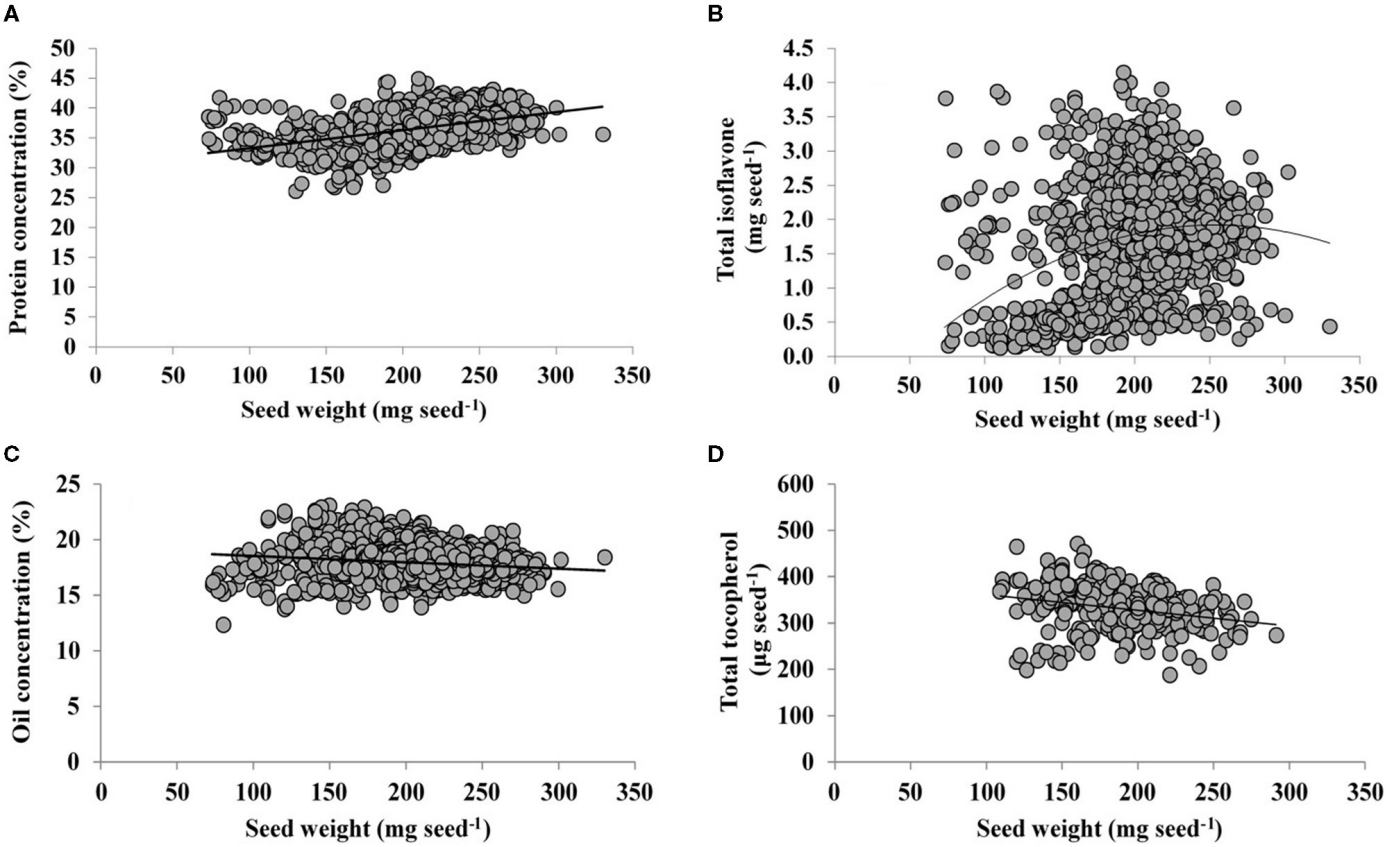
Relationship of seed protein concentration (%, adjusted to 13% moisture content) **(A)**, total isoflavone (mg seed^−1^) **(B)**, oil concentration (%, adjusted to 13% moisture content) **(C)**, and total tocopherol (μg seed^−1^) with seed weight. The line represents a fitted linear model for samples from a range of soybean genotypes and environments. The following models were computed: **(A)** *Y* = 30.2 + 0.03 *X*, adjusted *R*^2^ = 0.21, *P* < 0.0001; **(B)** *Y* = - 1.02 + 0.02*X* −5E-05 *X*^2^, adjusted *R*^2^ = 0.08, *P*_*X*_ < 0.0001, *P*_*X*2_ < 0.0001; **(C)** *Y* = 19.1–0.01*X*, adjusted *R*^2^ = 0.03, *P* < 0.0001; **(D)** *Y* = 396–0.34*X*, adjusted *R*^2^ = 0.06, *P* = 0.0001.

Final individual seed weight is a useful variable integrating physiological responses, with changes in seed weight closely related to variations in seed components. Indeed, across a range of environments, growing seasons, and genotypes, protein and seed weight were positively and linearly related (Figure 3A), with a slope of 0.03% protein mg seed^−1^. Interestingly, a quadratic model was fitted for TI content and seed weight (Figure 3B), indicating that TI rate increases less proportionally with increments in seed weight (0.02 mg seed^−1^ increase in TI up to 91 mg seed^−1^ then decreases steadily until a seed weight of 258 mg seed^−1^). From a similar range of seed weight, TI is diluted due to a lower deposition (Figure 3B) relative to protein (Figure 3A). On the other hand, responses of both oil concentration and TT content to seed weight were both linear and negative (Figures 3C,D), with a slope of −0.01 % oil and −0.34 μg TT seed^−1^ mg seed^−1^, respectively. Thus, although both traits decreases with increasing values of seed weight, TT decreases at a lower rate than oil, with both traits exhibiting dilution with large seed weights.

## Discussion

Broadly variation in soybean seed nutraceuticals relative to protein and oil components revealed that negative associations might not imply a detrimental effect on the synthesis of those compounds. The latter suggests that in some cases physical or genetic limitations at seed level may exist, altering the seed deposition rate, plausible playing a key role for understanding the physiological basis of the directions of the interrelationships between seed traits. The main goal of the current synthesis-analysis was to provide a more holistic interpretation of soybean seed nutraceuticals linked to agronomically relevant seed composition traits (e.g., oil, protein) under the umbrella of broad genotypic and environmental combinations. Although several studies presented associations among different seed components, those associations might be study-specific limited to those conditions with a narrow level of inference. In addition, one of the main roadblocks highlighted by this review is the lack uniformity of reporting units, lack of data on the interrelationships between nutraceutical and agronomically relevant seed traits, and lastly, a lack of proper context for linking changes in concentration without considering the relevancy of individual seed weight. These were the most critical elements needing to receive full attention for researchers, agronomists, breeders, and stakeholders investigating this seed quality topic in the near future.

From an agronomical seed composition standpoint, ranges in oil were within the spectrum reported in the scientific literature, from 13 to 24% (1, 27, 39, 60). On the other hand, even when the protein range explored in the current study was 2-fold greater (from 26 to 45%) than those reported in published scientific reports [i.e., 30–42%; e.g., Piper and Boote (27); Dardanelli et al. (60)], this range fell within values reported for some international soybean germplasm collection that informed 30 to 50%, like the USDA (1, 39). In terms of nutraceuticals, maximum TI content has been previously reported in field studies (ca. 4.0 mg seed^−1^) (16, 61) and for TT content, the maximum value documented in our dataset was 1.3- and 3.7-fold greater than the maximum reported by ILSI (61) and Ujiie et al. (62) (363 and 128 μg seed^−1^, respectively). Thus, the range of soybean seed quality compounds gathered in the current database was ample enough for analyzing relationships between nutraceutical composition with protein, oil, and seed weight.

Overall the present synthesis-analysis showed that TI was positively related to protein concentration, but at protein concentrations above 35% (13% moisture basis) increases in protein seem to be not proportionally accompanied by increments in seed TI. Indeed, Charron et al. (24) only observed a positive correlation of TI with protein in 5 out of 17 soybean cultivars, when protein percentage was below 35%. We found that for protein values above 35% TI increments decreased, highlighting the relevance of a more holistic approach when analyzing relashionships between seed components, since several studies in the literature that reported a trade-off between these traits, analyzed always datasets with protein levels above 35% (23, 28, 29, 63, 64). On the other hand, the relationship between TT-to-oil concentration was also positive as previously shown in the literature (65–67) suggesting an universal relationship between both variables. Only negative relationship for TT-to-oil was reported when evaluating low linolenic oil modified genotypes (19), which might responds indirectly to the reported positive correlation between linolenic and TT (68), but not being representative of commercial cultivars.

Previous investigations studied associations between seed traits by making correlation analyses that does not define basis of the associations and potentially implying cause-effect from highly correlated variables (69). In the present study, the boundary analysis by using quantile regression enabled us to obtain more meaningful agronomic and physiological conclusions. Although this analysis has been extensively used for studying relationships between resource availability and seed yield (33, 35, 57, 70), it has not been used for addressing changes in seed composition. For instance, comparing TI-to-protein concentration and TI-to-protein content relationships, the analysis showed that increases in TI accumulation were lower in the second case, suggesting that physical limits may exist for TI increments especially in the range of high protein level. Regarding oil content, the present synthesis-analysis showed non-significant relationship between TT and oil seed content. Nolasco et al. (71) in sunflower (*Helianthus annuus* L.) found a positive relationship between mentioned traits, but it is important to highlight that oil content in sunflower seeds is 3-fold than in soybean (72), and thus, more variation in oil content and a closer relationship between TT and oil content is expected. Then, taking into account the narrow variation of TT across the range of oil, it seems evident that there would be limited opportunity for further increasing TT content in soybean seeds.

Several studies have reported variations in soybean seeds isoflavones (10, 13, 16, 29, 73–77) as well as tocopherols (12, 18, 65, 67, 78). Nonetheless, many of those studies rarely addressed interactions between these nutraceuticals components with agronomically relevant traits such as protein, oil, and seed weight. Other major issue is that frequently, seed protein and oil are quantified in terms of their concentrations and not their contents, providing little or null insight into the physiological mechanisms underlying seed quality metabolism (79). Thus, the inclusion of seed weight is key for accounting variations in seed quality traits, taking into account that both protein and oil accumulation are relevant processes during the seed filling period. The present synthesis-analysis showed that when seed weight was above ca. 258 mg seed^−1^, the accumulation of TI per unit of seed weight was less than proportional; however, protein concentration continued increasing above this seed weight. From the oil perspective, both TT and oil concentration presented limitations to the increases in their accumulations as seed weight increases. This suggests that both TT and oil concentration are maintained as seed weight increased, suggesting that both components are smoothly diluted, whereas protein concentration can still accumulate (concentrate) as seed weight increased. However, TI showed a clear dilution.

The evident dilution of TI proposes a biological limitation to the accumulation of this nutraceutical component, not related to a physical restriction (i.e., seed size). Instead, environmental conditions during seed development and genetic factors such as gene linkage or pleiotropic effects (i.e., trait-by-trait interactions) could be underlying processes of this limitation at the biosynthesis-level (45, 63, 64, 80). Rotundo et al. (81) documented increases in seed size associated with increased protein and reduced oil concentrations. On the other hand, Nolasco et al. (71) for sunflower and Izquierdo et al. (30) for soybean, reported that TT content increased in a lower proportion than oil weight per grain. In the whole range of the explored seed weight, which was within that reported for the USDA soybean germplasm collection [40–340 mg seed^−1^, Nelson and Wang (82)] we observed that as seed weight increased, TT decreased at a higher rate than oil concentration. The lack of association between TT-to-oil content in our synthesis-analysis could be pointing toward compensatory effects of opposite total tocopherol content responses to oil concentration and seed weight, respectively.

**Figure 4:**
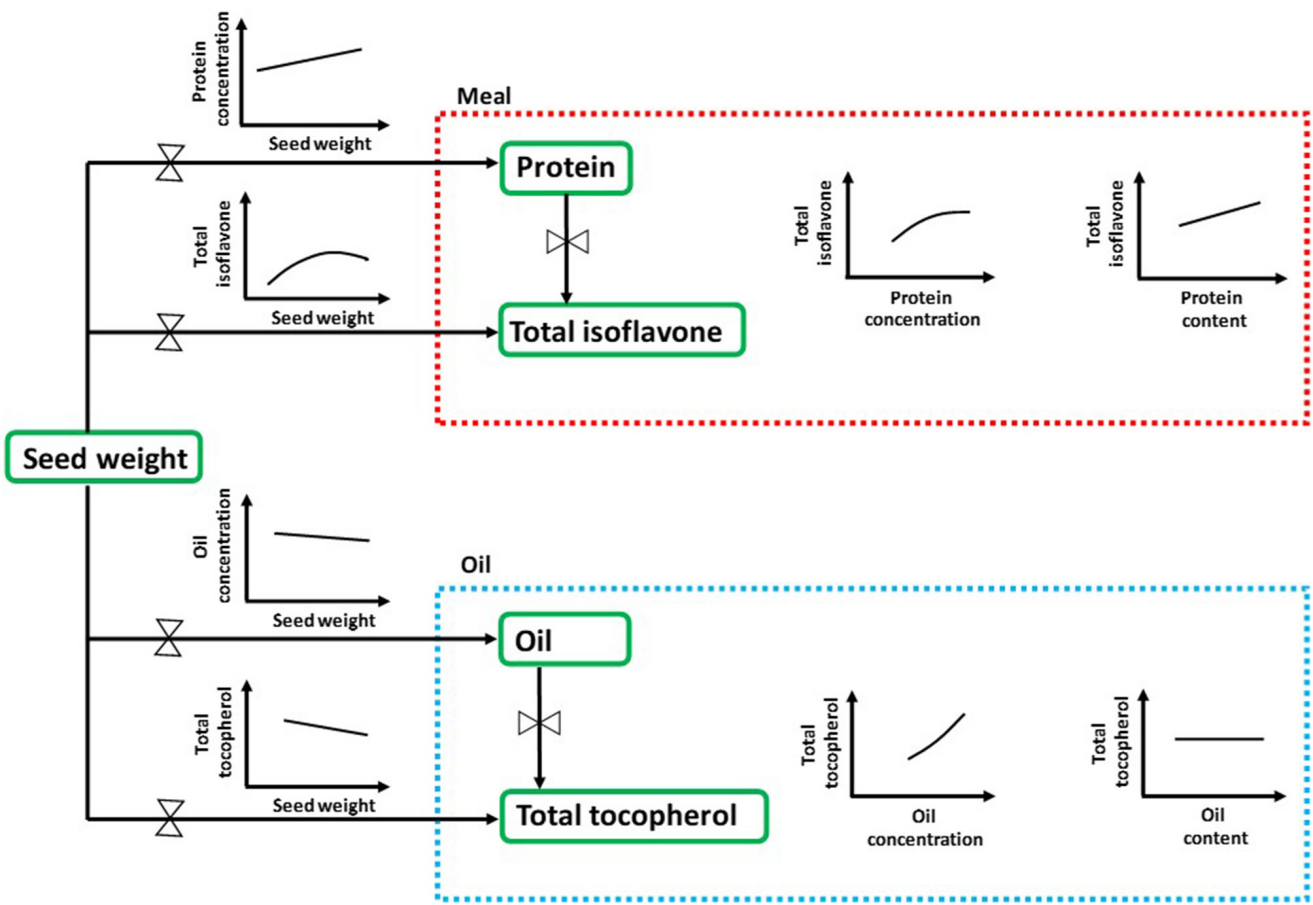
Comprehensive scheme of quality matrix highlighting the main interrelationships between seed nutraceuticals in soybean with the major seed components concentrations and contents through an ecophysiological perspective. Protein and oil concentrations are expressed as percentage of dry weight, whereas contents are expressed as mg component per seed on a dry basis. Total isoflavone and total tocopherol contents are expressed as mg and μg component per seed on a dry basis, respectively.

Seed chemical composition is the result of complex interactions among seed genetic characteristics and the environment (83). In the current study, we have provided a comprehensive and effective approach for understanding the natural variation of seed nutraceuticals in soybean with an ecophysiological perspective, i.e., analyzing their interrelationships with the major seed components concentrations and contents (Figure 4). Seed weight, closely linked to seed composition and often overlooked in many seed nutraceutical composition studies, arises as an important trait to be further investigated for addressing both seed industrial and nutraceutical quality. Furthermore, future research is needed in order to shed light into the physiological mechanisms occurring during the seed filling period to better understand the effect of environmental conditions (e.g., temperature, water and nutrient availability, radiation), genotype, and/or management practices on modulating changes in soybean seed nutraceuticals. Also, to bridge the gap between soybean matrix constituents, research should also advance toward the relationship between seed protein and carbohydrates. As it is well-documented, increases in protein with reductions in carbohydrates would contribute to enhancing the nutritional value of soybean meal (84, 85). The main outcomes presented in this synthesis-analysis provide for the first time, to extend of our knowledge, valuable practical data on the association between nutraceuticals and protein, oil, and seed weight for soybean crop. This review provides foundational knowledge for soybean breeding programs interested on predicting and selecting enhanced meal isoflavone and/or oil tocopherol contents and their relationships with less-cost intensive and more rapidly-assessed agronomically relevant seed traits such as protein and oil content.

## Author Contributions

CC, FS, and IC contributed to conception and design of the study, organized the database and performed the statistical analysis, and prepared and reviewed the manuscript. All authors contributed to manuscript revision, read, and approved the submitted version.

## Conflict of Interest

The authors declare that the research was conducted in the absence of any commercial or financial relationships that could be construed as a potential conflict of interest.

## Publisher's Note

All claims expressed in this article are solely those of the authors and do not necessarily represent those of their affiliated organizations, or those of the publisher, the editors and the reviewers. Any product that may be evaluated in this article, or claim that may be made by its manufacturer, is not guaranteed or endorsed by the publisher.

## Abbreviations

TI: total isoflavone
TT: total tocopherol.
